# Sheehan's Syndrome Revisited: Underlying Autoimmunity or Hypoperfusion?

**DOI:** 10.1155/2018/8415860

**Published:** 2018-02-26

**Authors:** José Gerardo González-González, Omar David Borjas-Almaguer, Alejandro Salcido-Montenegro, René Rodríguez-Guajardo, Anasofia Elizondo-Plazas, Roberto Montes-de-Oca-Luna, René Rodríguez-Gutiérrez

**Affiliations:** ^1^Endocrinology Division, Department of Internal Medicine, University Hospital “Dr. José E. González”, Universidad Autonoma de Nuevo Leon, 64460 Monterrey, NL, Mexico; ^2^Research Unit, University Hospital “Dr. José E. González”, Universidad Autonoma de Nuevo León, 64460 Monterrey, NL, Mexico; ^3^Gastroenterology Division, University Hospital “Dr. José E. González”, Universidad Autonoma de Nuevo León, 64460 Monterrey, NL, Mexico; ^4^Gynecology and Obstetrics Division, University Hospital “Dr. José E. González”, Universidad Autonoma de Nuevo Leon, 64460 Monterrey, NL, Mexico; ^5^Histology Department, Facultad de Medicina, Universidad Autonoma de Nuevo Leon, 64460 Monterrey, NL, Mexico; ^6^Knowledge and Evaluation Research Unit in Endocrinology, Mayo Clinic, Rochester, MN 55905, USA; ^7^Division of Endocrinology, Diabetes, Metabolism and Nutrition, Department of Medicine, Mayo Clinic, Rochester, MN 55905, USA

## Abstract

Sheehan's syndrome remains a frequent obstetric complication with an uncertain pathophysiology. We aimed to assess the incidence of hypopituitarism (≥2 hormonal axis impairment) within the first six postchildbirth months and to determine the existence of anti-pituitary antibodies. From 2015 to 2017, adult pregnant women, who developed moderate to severe postpartum hemorrhage (PPH), were consecutively included in the study. Pituitary function was assessed 4 and 24 weeks after PPH. At the end of the study, anti-pituitary antibodies were assessed. Twenty women completed the study. Mean age was 26.35 (±5.83) years. The main etiology for severe PPH was uterine atony (65%) which resulted mostly in hypovolemic shock grades III-IV. Within the first four weeks after delivery, 95% of patients had at least one hormonal pituitary affected and 60% of the patients fulfilled diagnostic criteria for hypopituitarism. At the end of the study period, five patients (25%) were diagnosed with hypopituitarism (GH and cortisol axes affected). Anti-pituitary antibodies were negative in all patients. At 6 months follow-up, one in every four women with a history of moderate-to-severe PPH was found with asymptomatic nonautoimmune-mediated hypopituitarism. The role of autoimmunity in Sheehan's syndrome remains uncertain. Further studies are needed to improve the remaining knowledge gaps.

## 1. Introduction

Sheehan's syndrome remains a frequent obstetric complication in emergent and developed countries that to date still reports a relatively high prevalence of moderate to severe postpartum hemorrhage (PPH) [1–4]. Sheehan's syndrome has been usually described to affect pregnant woman after moderate to profound hypovolemic shock throughout delivery. However, it is usually diagnosed months to years after the hemorrhagic event [5, 6]. Due to its delayed diagnosis, clinical presentation (which usually impairs quality of life), and potentially life-threatening complications (e.g. coma or death), Sheehan's syndrome still remains important to pregnant women, clinicians, and public health services around the world [6].

The pathophysiology of Sheehan's syndrome has been classically attributed to a transient hypoperfusion that provokes infarction, necrosis, and consequent dysfunction in a physiologically enlarged pituitary gland (due to pregnancy) [5, 7–9]. The next rational pathophysiological step would be an immediate hypopituitarism; however, this is rarely the case. De facto, Sheehan's syndrome's reported incidence in patients, who suffered PPH, ranges from 0 to 30% [10–12]. Previous studies that have prospectively assessed pituitary function shortly after a PPH event have had small sample size or a short follow-up period. Consequently, their results are difficult to interpret [10, 11]. Moreover, not every woman who suffers PPH develops Sheehan's syndrome and when they do, it manifests within a wide spectrum of time (from months to years), suggesting that there are other factors that influence its appearance. Recently, several studies have assessed the role that autoimmunity could have in the pathophysiology of Sheehan's syndrome [13–16]. De Bellis et al. retrospectively detected anti-hypothalamic antibodies and anti-pituitary antibodies in the serum of patients diagnosed with Sheehan's syndrome (40% and 35%, resp.) [15]. However, because these autoantibodies were found years after the disease was established, their role in the pathophysiology of Sheehan's syndrome remains uncertain.

Consequently, we decided to conduct this prospective study in patients who suffered moderate to severe PPH with the primary objective of assessing the incidence of hypopituitarism within the first six months postchildbirth and to determine the existence of anti-pituitary antibodies. Secondary endpoints were to correlate clinical variables with hemorrhage intensity and pituitary dysfunction.

## 2. Patients and Methods

### 2.1. Patient Population

After the Institutional Review Board and Ethics Committee from our University approved the study protocol, we began recruitment. Written informed consent was obtained from all participants before enrollment. Women ≥ 18 years old who developed PPH in the Obstetric Unit of the University Hospital “Dr. Jose E. Gonzalez” were consecutively invited to participate in the study. PPH was defined as having one or more of the following criteria: (a) postvaginal partum blood loss ≥ 1000 ml, (b) postcesarean partum blood loss ≥ 1500 ml, (c) hypovolemic shock grade III or IV, (d) hysterectomy due to unstoppable bleeding, and (e) hemoglobin decrease ≥ 3 g per liter immediately after delivery. Patients with a past medical history of any thyroid disease, suprarenal abnormalities, pituitary malfunction, or active tuberculosis were excluded from the study.

### 2.2. Study Protocol

All patients were recruited at bedside immediately (≤3 hours) after the PPH event. A complete medical clinical history and baseline anthropometric measures were assessed. Data regarding the PPH's characteristics as well as the newborns' vital signs and APGAR scores were also documented. Because of the association between hypopituitarism and diabetes insipidus, monitoring of the liquids' input and output was closely followed during their hospitalization period. Before their discharge, patients were instructed to look for symptoms related to hypopituitarism as well as diabetes insipidus. On follow-up, patients were reassessed four and 24–28 weeks after the PPH event in order to undergo clinical assessment and pituitary dynamic testing. Between visits, patients were contacted via telephone every four weeks in order to detect the appearance of symptoms related to hypopituitarism (e.g., agalactia, amenorrhea, impaired mental status, and fatigue) and diabetes insipidus (e.g., thirst and an excessive amount of urine). Hypopituitarism was diagnosed when two or more pituitary hormonal axes were impaired. Enhanced magnetic resonance imaging (MRI) of the pituitary gland was obtained from patients diagnosed with hypopituitarism. Anti-pituitary antibodies were assessed at the end of the study.

### 2.3. Measurements

Clinical data regarding pregnant women and newborns' vital signs, APGAR score, hemorrhage quantification, infused solutions, and red blood cell units transfused at the time of the event were taken from a secured medical record. On every follow-up visit, patients' weight and height were determined on a calibrated Seca 700 scale and stadiometer (TAQ Sistemas Médicos, S.A. de C.V., Mexico City, Mexico), respectively, in order to calculate insulin dosage for the dynamic pituitary testing (0.1 IU of regular insulin per kilogram).

#### 2.3.1. Pituitary Dynamic Tests

After an eight-hour fast, blood samples were drawn to obtain baseline glucose, cortisol, thyroid-stimulating hormone (TSH), free T4, total T3, total T4, growth hormone, prolactin, follicle-stimulating hormone (FSH), luteinizing hormone (LH), and estradiol determinations. Additional blood was centrifuged, and the obtained serum was stored in aliquots and frozen at −20°C for later processing and autoantibody detection. Next, we administrated leuprolide (100 *μ*g) to stimulate FSH and LH secretion, TRH (200 *μ*g) to stimulate TSH and prolactin secretion, and regular insulin 0.1 IU/kg to trigger hypoglycemia (glucose ≤45 mg/dl) and stimulate growth hormone (GH) and ACTH/cortisol secretion. Blood samples were taken at minutes 0, 15, 30, 60, and 90 after the triple bolus administration. If the glucose levels failed to decrease below 45 mg/dl with the first insulin dose, a second dose was administrated. All samples were frozen and assessed twice at the same time at the end of the study. The glucose oxidase method was used to assess fasting plasma glucose (Stat-Fax Spectrophotometer, Awareness Technology, Palm City Fl.); intra-assay and interassay coefficients of variation (CV) were 1.4% and 0.6%, respectively. FSH, LH, prolactin, TSH, total T4, free T4, and cortisol were measured using electrochemiluminescence in a Cobas® 6000 e601 analyzer series (Roche, Germany). Intra-assay and interassay CV for the different hormones were 2.8% and 4.5%, respectively, for FSH, 1.2% and 2.2%, respectively, for LH, 1.7% and 2.0%, respectively, for prolactin, 3% and 7.2%, respectively, for TSH, 1.8% and 4.2%, respectively, T4, 5% and 6.3%, respectively, for free T4, 1.7% and 2.8%, respectively, for cortisol. GH and total T3 were measured using the electrochemiluminescence method in an IMMULITE® 1000 Immunoassay System (Siemens, Germany); intra-assay and inter-assay CV were 6.5% and 6.2%, respectively, for GH and 3.9% and 5.3%, respectively, for T3.

A normal response to the pituitary dynamic test was considered as follows: (a) a >2-fold and a >1.5-fold increase for LH and FSH levels, respectively, (b) a peak TSH of >2.5-fold or a >5 mU/l increase in addition to normal free T4 levels (>0.7 ng/dl), (c) a 2.5-fold increase in serum prolactin levels, (d) a peak cortisol response of >18 *μ*g/dl or a >5 *μ*g/dl increase, and (e) a GH peak of >5 *μ*g/l. If two or more axes were impaired, hypopituitarism was diagnosed [17]. A diagnosis of secondary hypothyroidism was made in the absence of 2.5-fold increase or >5 *μ*U/l increase in TSH levels [17, 18]. Likewise, we considered that prolactin secretion was altered if the pituitary dynamic tests' normality criteria were not met [17].

#### 2.3.2. Detection of Anti-Pituitary Antibodies by Western Blot

Anti-pituitary antibodies were determined by Western blot. A total of 15 patients with previously confirmed Sheehan's syndrome and five with lymphocytic hypophysitis donated serum samples, which were used as positive controls. Likewise, serum samples of 10 healthy men and women not related to the study participants were used as negative controls. All samples were assessed twice and at the same time at the end of the planned follow-up. Human pituitary protein lysates and gamma enolase recombinant protein were used as target antigens for anti-pituitary antibody detection. Human embryonic kidney (HEK-293) cell protein lysates were used as negative control.

HEK-293 cell line (#CRL-1573) was obtained from the American Type Culture Collection (Manassas, VA, USA). HEK-293 cells were cultured in advanced Dulbecco's modified Eagle medium supplemented with 4% heat-inactivated fetal calf serum, 2 mM L-glutamine, and 100 U/ml penicillin/streptomycin (all from Cellgro, Mediatech Inc., Manassas, VA, USA). Human pituitary samples were donated by the Forensic Department of our University and were collected following institutional guidelines and snap frozen in liquid nitrogen. Subsequently, human pituitary samples and HEK-293 cells were processed with ProteoJET Mammalian Cell lysis Reagent (#K0301; Fermentas, Waltham, MA, USA) according to the manufacturer's instructions. The extracted HEK-293 cells and human pituitary protein samples as well as gamma enolase recombinant proteins (#N2175; US Biological, Salem, MA, USA) were subjected to denaturing electrophoresis in 12% SDS-PAGE gels. Proteins were transferred to PVDF membranes and then blocked for 1 hour with 10% bovine serum albumin (BSA) (#A1311; US Biological, Salem, MA, USA) in Tris-buffered saline and Tween 20 (TBST) (135 mM NaCl, 2.7 mM KCl, 24.8 mM Tris-HCl, 0.05% Tween 20, pH 7.4).

Afterwards, patients' serum and controls' serum were diluted at 1 : 50 concentration in 0.3% BSA in TBST buffer solution. The diluted serum samples were incubated with the membranes overnight. The membranes were washed with TBST and incubated for 2 hours with a horseradish peroxidase-conjugated goat anti-human IgG antibody (sc-2453; Santa Cruz Biotechnology Inc., Dallas, TX, USA) diluted at 1 : 5000. The gamma enolase recombinant protein was detected with a mouse anti-*γ* enolase antibody (sc-21738; Santa Cruz Biotechnology Inc., Dallas, TX, USA) diluted at 1 : 500 and then incubated for two hours with a horseradish peroxidase-conjugated rabbit anti-mouse antibody (#A9044; Sigma-Aldrich; Merck Millipore) diluted at 1 : 10,000. Signal detection was performed using Super Signal West Pico Chemiluminescent Substrate (#34080; Pierce Biotechnology; Thermo Fisher Scientific Inc.), and the membranes were scanned on a C-DiGit Scanner Model 3600 (Li-cor, Lincoln, NE, USA).

### 2.4. Imaging Study

Enhanced MRI of the pituitary gland was performed in a General Electric Sigma Excite 1.5T MR scanner (GE Medical Systems). Two experienced neuroradiologists, separately and independently, interpreted the images. Empty sella (complete or partial) and any other structural lesions that could explain hypopituitarism were particularly looked for.

### 2.5. Statistical Analysis

Continuous variables are reported as means and standard deviation. Categorical variables are reported as percentages and frequencies. Normality was studied using the Shapiro Wilk test. Student's *t*-test and the Mann–Whitney *U* test were used to compare continuous variables according to normality. Categorical variables were compared using a Pearson's *χ^2^* test or Fisher's exact test for 2 × 2 tables. A *P* value ≤ 0.05 was considered statistically significant. Patients who failed to complete follow-up were not analyzed. The statistical analysis was performed using IBM SPSS Statistics 20.0 (IBM Corp., Armonk, NY).

## 3. Results

### 3.1. Study Population

Between March 2015 and January 2017, a total of 23 patients were included in the study. Of these, 20 women (86.7%) completed the study. Of the three women that left the study, two were lost to follow-up for no clear reason (despite efforts to contact them) and one was excluded due to a new pregnancy.  Table 1 shows the overall patients' baseline characteristics. Mean age was 26.35 (±5.83) years. The main etiology for severe PPH was uterine atony (65%) followed by vaginal tearing (15%). Most patients presented at least grades III-IV of shock (75%). There were no differences, in any of the baseline clinical characteristics, between patients with hypopituitarism and normal pituitary function.

### 3.2. Dynamic Pituitary Tests

Within the first 4 weeks after delivery, 19 out of 20 patients (95%) had at least one hormonal pituitary axis affected and 60% (12/20) of the patients fulfilled the diagnosis criteria for hypopituitarism. At the time of their second and final visit (6 ± 0.2 months), patients who remained with at least one affected axis was reduced to 56% (11/20). At the end of the study period, five out of 20 patients (25%) fulfilled the criteria for hypopituitarism. In consequence, between their first and second visit, pituitary dynamic testing returned to normal in 35% of patients. The comparison of each pituitary axis is described in Table 2.

Almost every patient who presented alteration of the FSH/LH axis on the first visit fully recovered at the second visit (8/9). Of the patients who presented alteration in the GH axis on their first results, 26.6% had normal results on their second visit (4/15). A total of 75% of the patients with prolactin secretion impairment recovered its functionality on their second visit. The patient with the TSH axis dysfunction remained altered on the second visit. Finally, of the five patients with cortisol dysfunction at their first visit, three improved at their second assessment. Moreover, cortisol dysfunction was addressed in another three patients, ending with a total of five patients with ACTH axis deficiency.

When comparing mean peak hormonal axes values between the first and second hormone assessment, only cortisol and GH had significant statistical differences between the groups (*P* < 0.0001 and *P* = 0.019, resp.) (data not shown). After a detailed clinical history, none of the women described any symptoms related to hypopituitarism and every physical examination was unremarkable.

### 3.3. Clinical Factors Associated with Pituitary Dysfunction Persistence after 6 Months

When comparing the baseline characteristics of the event, there were no statistically significant risk factors associated with pituitary dysfunction development. However, a lower, but not significant, systolic blood pressure at the time of hypovolemic shock was seen in patients with persisting pituitary dysfunction (77.6 ± 17.28 versus 100.6 ± 24.63 mmHg, *P* = 0.07) (Table 1).

### 3.4. Anti-Pituitary Antibodies

**Figure 1:**
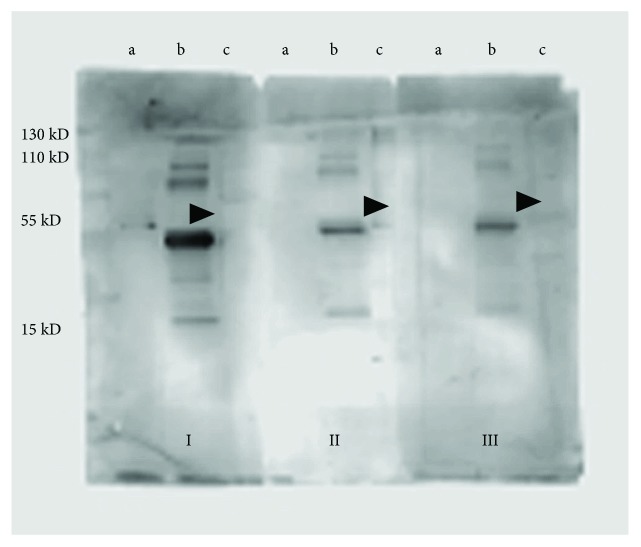
Representative Western blot of human pituitary extracts. Serum samples from patients 16 and 20 (I, II) and healthy subjects (III) were tested on recombinant gamma enolase (a), protein lysates from human pituitary (b), and HEK-293 cells (c). Arrowheads indicate a nonspecific protein band of about 55 kDa which was seen on all lanes with human pituitary's protein lysates, from both patients and healthy subjects. MW: molecular weight marker.

Serum from patients and healthy subjects were tested on a PVDF membrane that contained recombinant gamma enolase and protein lysates from human hypophysis and from HEK-293 cells. In neither patients nor controls, specific antibodies that recognized protein bands were not detected, implying the absence of anti-pituitary antibodies (Figure 1).

A nonspecific protein band of about 55 kDa was detected on all the lanes with human pituitary's protein lysates from both patients and healthy subjects, which may correspond to immunoglobulins present in the pituitary's lysate sample.

### 3.5. Imaging Study

The five patients who fulfilled the criteria for hypopituitarism underwent an enhanced MRI of the pituitary gland two weeks after their second dynamic pituitary test. None of the MRI studies revealed structural alterations that could explain the patients' altered pituitary function.

## 4. Discussion

In this prospective study, at six months follow-up, five out of 20 pregnant women with PPH were found to have hypopituitarism. Interestingly, all patients were asymptomatic and anti-pituitary antibodies were negative in all. In addition, clinical characteristics and PPH factors were not found to be associated with the risk of patients developing Sheehan's syndrome. To our knowledge, this is the largest prospective study evaluating women's risk of developing Sheehan's syndrome.

A few weeks after PPH, a higher proportion of hypopituitarism (60%) was observed; however, only 25% of the patients ended the study with hypopituitarism. This suggests that after the event, there was a transient period of subclinical pituitary dysfunction in 35% of the patients. In our study and consistent with previous data, the GH axis was the most frequent axis impaired (10/23). Interestingly, almost every patient with FSH/LH axis dysfunction during the first four weeks recovered at the end of follow-up (eight out of nine). However, only one third of the women with impaired GH axis recovered (5/15). Even though there are several hypotheses for this observation, the pathophysiology behind it remains unclear; however, it is important to acknowledge that FSH, LH, and GH have been reported to physiologically decrease during pregnancy [19]. Of note, only the mean peak GH and cortisol concentrations during the second visit were different among patients with hypopituitarism. Given that GH was the most frequently impaired pituitary axis and cortisol usually increases during pregnancy, a low basal cortisol seems to predict its stimulated abnormality. Consequently, these two pituitary axes seem to be the most important to assess in patients with moderate to severe PPH.

Our results showed that after 6-7 months, the pituitary impairments present during the first four weeks after delivery veered to improve in the majority of patients (58.33%). In fact, there have been some case reports where patients with Sheehan's syndrome recover back to normal less than 1 year after the event [20, 21]. Nevertheless, because pituitary function impairment in Sheehan's syndrome can manifest after several years, some of our patients may experience the appearance of symptoms in future years [6, 22–24]. Our findings raise even more questions about the pathophysiology behind Sheehan's syndrome as it increases the confidence in the fact that hypoperfusion is not enough per se to rationally explain the pathophysiology of the disease. Of note, all women in our study were asymptomatic and were not receiving corticosteroids or any other hormonal supplementation therapy before and during the study was carried out. While the follow-up period was probably not long enough for the development of symptoms, our findings suggest that new recommendations, which may include screening for pituitary dysfunction (with particular emphasis on GH and cortisol axes) within the first six months after a moderate to severe PPH, might be needed. Moreover, previous studies had reported that some patients with Sheehan's syndrome present diabetes insipidus [25]. Nevertheless, none of the patients in our study presented symptoms or laboratory findings suggestive of diabetes insipidus during the study follow-up period (data not shown).

Recent studies suggest that autoimmunity plays a major role in the pathophysiology of Sheehan's syndrome. In fact, De Bellis et al. found that an important proportion of Sheehan's syndrome patients (between three and 40 years of evolution) had anti-pituitary and anti-hypothalamic antibodies (35% and 40%, resp.). Despite these findings, it is unclear if anti-pituitary and anti-hypothalamic antibodies are the cause, enhancers, consequence of the disease, or silent confounders. In our study, the follow-up was more than enough for the appearance of autoantibodies; however, no anti-pituitary antibodies were documented in any patient, neither at four weeks nor during the following months after delivery. This suggests that, in any case, autoantibodies in Sheehan's syndrome behave more as an enhancer of the disease rather than an etiology factor. However, because of the natural course of the disease, it is still plausible that autoantibodies may be found many months or years after the antigen exposition to the immune system and perpetuate the hypopituitarism dysfunction, as previously reported [15, 22–24]. Although Sheehan's syndrome often presents structural damage such as complete or partially empty sella, we found a completely normal MRI in all the patients [6, 22, 23]. We hypothesize that no structural alterations were observed because the MRI evaluation in our study population was done earlier in the course of the disease. This should be assessed in future follow-up studies.

There are several limitations to our study. First, the follow-up period was probably not enough to bring to light the classical signs and symptoms of hypopituitarism. However, we think this is also a strength of the study due to the fact that a prompt diagnosis enables on-time treatment that can be offered to the patient before clinical manifestations of hypopituitarism impair their quality of life and potentially threaten it. In addition, we will continue to follow in the next years this cohort and will be able to report in the future their biochemical and clinical behaviors. Second, IGF-1 and ACTH values were not measured in this study. This could generate doubts about the origin (i.e., primary or secondary) of the impairments found. Nevertheless, because of the nature of the disease, we considered that their measurements were not necessary. However, we did use the gold standard stimulation test (insulin-mediated hypoglycemia) for both GH and cortisol. Third, there was a lack of a control group in the study; however, there is strong data supporting the notion that pituitary function remains unaltered during an uncomplicated delivery. Fourth, probably, a larger sample size and particularly a higher number of hypopituitarism events could help uncover risk factors that can predict the risk of hypopituitarism. However, our cohort is representative of the average in our community, it is the largest prospective study reported of its kind, and assessment of exposure was through the use of secured medical records. Finally, while it is plausible that other antibodies not measured in our study may play a role in the genesis of Sheehan's syndrome, to our knowledge, for the first time in a prospective study, we assessed the most common anti-pituitary antibodies described in the body of evidence.

## 5. Conclusion

In this prospective study of women with moderate to severe PPH, one out of four was found to have, at six months follow-up, asymptomatic nonautoimmune-mediated hypopituitarism. Almost two-thirds of the patients with hypopituitarism during the first four weeks returned to normal at the end of the study. None of the baseline clinical characteristics was found to be associated with an increased risk of hypopituitarism. Further studies are needed to increase our knowledge in the pathophysiology and presentation of this illness that still affects a considerable number of women and impairs their health and quality of life.

## Figures and Tables

**Table 1:** Demographic characteristics and clinical variables.

Characteristic	Total	Hypopituitarism	Normal test	*P* value
Patients, *n* (%)	*n* = 20	*n* = 5	*n* = 15	
Age (years), mean (SD)	26.35 (±5.83)	26.40 (±5.94)	26.33 (±6.01)	0.951
Menarche (years), mean (SD)	11.9 (±1.20)	12.40 (±1.14)	11.73 (±1.22)	0.298
Previous hemorrhage, *n* (%)	1 (5)	0 (0)	1 (6.7)	0.554
Gravida, *n* (%)				
1	7 (35)	0 (0)	7 (46.7)	0.077
2	6 (30)	3 (60)	3 (20)	
≥3	7 (35)	2 (40)	5 (33.3)	
Cesarean section, *n* (%)	9 (45)	1 (20)	8 (53.3)	0.319
Weeks of gestation, mean (SD)	38.16 (±2.29)	38.56 (±1.44)	38.02 (±2.56)	0.745
Hemorrhage etiology, *n* (%)				
Uterine atony	13 (65)	3 (60)	10 (66.7)	0.285
Preeclampsia/eclampsia	1 (5)	0 (0)	1 (6.7)	
Uterine rupture	1 (5)	1 (20)	0 (0)	
Placenta previa	2 (10)	1 (20)	1 (6.7)	
Vaginal tearing	3 (15)	0 (0)	3 (20)	
Hysterectomy, *n* (%)	10 (50)	4 (80)	6 (40)	0.303
Hemorrhage (ml), mean (SD)	2070 (±1130.95)	2420 (±1247.79)	1953.33 (±1110.25)	0.439
Infused solutions (ml)	3363.15 (±1754.74)	4290 (±2237.85)	3032.14 (±1508)	0.176
RBC units (500 ml each), mean (SD)	3.4 (±2.7)	4.6 (±3.3)	3.1 (±2.5)	0.284
Shock grade, *n* (%)				
2	5 (25)	0 (0)	5 (33.3)	0.157
3	6 (30)	3 (60)	3 (20)	
4	9 (45)	2 (40)	7 (46.7)	
Shock index, mean (SD)	1.39 (±0.56)	1.74 (±0.53)	1.27 (±0.54)	0.113
Systolic BP, mean (SD)	94.9 (±24.79)	77.6 (±17.28)	100.66 (±24.63)	0.07
Diastolic BP, mean (SD)	58.1 (±18.00)	54.4 (±20.16)	59.33 (±17.81)	0.825
Heart rate, mean (SD)	120.65 (±20.60)	128.2 (±12.49)	118.13 (±22.45)	0.358
Time to stability (minutes), mean (SD)	74.21 (±28.34)	88.75 (±38.16)	70.33 (±25.38)	0.26
Hb preevent, mean (SD)	12.13 (±1.25)	12.1 (±0.58)	12.15 (±1.42)	0.938
Post Hb, mean (SD)	8.92 (±1.90)	10 (±2.13)	8.56 (±1.74)	0.148
Delta Hb, mean (SD)	−3.217 (±2.10)	−2.1 (±2.12)	−3.58 (±2.02)	0.119

RBC: red blood cell; BP: blood pressure; Hb: hemoglobin.

**Table 2:** Pituitary dynamic test results.

Patient number	TSH (*μ*U/l)			Prolactin (ng/ml)		LH (*μ*U/l)		FSH (*μ*U/l)		Cortisol (*μ*g/dl)		GH (*μ*g/l)	
	Basal	Highest value	Free T4 (ng/dl)	Basal	Highest value	Basal	Highest value	Basal	Highest value	Basal	Highest value	Basal	Highest value
First visit													
1	1.97	2.53^∗^	0.77	11.22	24.07^∗^	0.17	0.17^∗^	<0.1	<0.1^∗^	10.81	17.58	1.81	1.91^∗^
2	2.4	18.02	0.89	321.9	>470	0.15	0.17^∗^	<0.1	0.19^∗^	13.46	21.18	0.97	0.76^∗^
3	1.15	10.39	1.2	12.94	159	4.84	59.33	4.61	16.08	15.63	11.62^∗^	4.13	0.99^∗^
4	1.38	18.92	0.99	61.1	>470	0.19	0.23^∗^	0.11	0.75	23.74	40.25	0.15	0.88^∗^
5	0.96	5.27	1.03	21.61	137.1	2.62	26.22	4.76	15.32	12.56	16.46^∗^	7.87	6.36
6	3.05	15.4	0.9	334.9	>470	0.26	0.22^∗^	0.13	0.16^∗^	39.35	51.51	0.95	3.81^∗^
7	1.15	11.78	1.09	155.9	269.6^∗^	0.2	0.21^∗^	0.1	0.18^∗^	30.35	40.27	1.42	18.3
8	5.3	28.8	1.07	228.6	>470	0.18	0.23^∗^	0.21	0.51	62.9	62.8	0.39	0.38^∗^
9	1.86	6.59	1.14	83.94	297	0.29	0.82^∗^	3.33	10.9	13.63	10.88^∗^	0.33	0.84^∗^
10	0.42	3.6	1.6	65.5	269.9	0.2	0.3^∗^	0.52	1.9	16.3	24.9	2.21	0.68^∗^
11	0.7	9.7	1.1	18.9	>470	0.22	0.35^∗^	1.6	4.1	15.1	13.8^∗^	<0.05	0.18^∗^
12	1.6	8.5	1.22	39.5	97.1^∗^	2.1	11.7	7.1	23.6	31.9	40.5	0.13	1.89^∗^
13	1.64	13.5	1.23	13.2	121.6	5.64	133.8	4.6	27.4	6.6	15.3	<0.05	0.42^∗^
14	1.7	11.3	1.39	80.1	>470	1.9	5.4^∗^	6.1	16.2	28.3	33.7	0.68	20.7
15	1.3	11.9	1.3	84.1	188	9.6	86.8	5.6	17.4	16.6	26.8	0.14	4.4^∗^
16	2.59	12.72	1.15	104	171.5^∗^	5.54	16.98	8.21	19.05	15.9	13.45^∗^	0.37	0.32^∗^
17	0.8	8.07	1.38	31.72	217.4	5.46	23.78	6.6	16	14.7	18.16	2.3	3.25^∗^
18	0.93	30.9	1.13	175.5	444.8	4.61	44.79	10.68	39.53	10.83	17.26	0.2	2.3^∗^
19	2.22	26.06	0.9	141.6	>470	<0.1	<0.1^∗^	0.49	1.49	18.9	37.4	0.42	5.69
20	2.2	14.1	1.06	11.6	120.2	3	112.7	3.9	29.5	22.4	24.9	3.64	21.4

Follow-up visit													
1	1.82	3.48^∗^	0.598	10.5	24.33^∗^	6.09	12.51^∗^	12.65	16.06	7.75	9.26^∗^	0.077	0.453^∗^
2	1.7	12.7	1.12	10	61.6	9.9	74.2	2.9	8.3	11.8	8.4^∗^	0.38	0.17^∗^
3	1.5	8.3	1.77	11.1	83.4	7.9	74.2	3.8	10.3	14.1	14.9^∗^	3.1	0.26^∗^
4	1.34	21.15	1.15	12.48	69.48	1.1	25.43	2.2	17.09	9.02	17.6	0.05	4.67^∗^
5	0.88	8.9	1.18	4.3	38.4	3.5	46.6	2.8	9.1	11.9	24.8	7.5	6.6
6	1.5	12.9	1.4	15.7	104.3	7.1	70.4	4.9	12.8	30.4	33.5	8.7	10.1
7	0.72	6.1	1.6	32.5	82.2	10.3	>200	2.9	27.6	38.9	39.1	9.1	8.1
8	2.9	17.9	1.3	66.6	241.6	24.6	137.9	6.5	14.6	11.3	25.3	2.9	15.5
9	3.3	21.1	1.26	18.3	244.9	10	116.8	5.9	25.7	26.7	25.5	12.3	9.5
10	1.3	10.74	1.45	15	88.7	3.99	82.62	1.71	5.77	13.1	23.6	7.22	17.2
11	2.52	23.45	0.94	13.49	99.85	3.51	70.65	6.73	28.9	17.19	29.59	<0.05	3.69^∗^
12	1.07	9.74	1.26	32.76	115.7	12.77	140.5	4.68	13.26	12.9	38.4	0.11	7.83
13	1.16	9.02	1.19	11.44	48.38	9.52	44.72	4.48	9.31	9.49	16.27	0.14	0.56^∗^
14	2.89	14.24	1.23	51.27	144.7	2.23	23.9	5.03	12.24	22.53	22.87	<0.05	0.1^∗^
15	1.54	17.57	1.16	97.41	332.5	4.13	53.31	2.25	5.61	9.22	11.61^∗^	2.21	2.23^∗^
16	15.25	>100	0.8	24.72	267.1	7.78	40.06	5.79	10.78	8.01	6.35^∗^	0.11	0.15^∗^
17	0.89	10.64	1.27	16.8	92.25	6.04	19.65	6.51	9.37	13.37	22.68	0.46	4.8^∗^
18	2.3	14.9	1.3	10.5	90.8	4.4	60	3.6	8.6	9.5	20.1	2.28	6.99
19	2.6	26.2	1.18	13.8	122.9	4.3	35.7	2.9	8.7	13.9	25.9	0.1	3.26^∗^
20	1.95	12.7	1.19	8.25	101.2	12.52	160.3	5.3	14.3	28.3	28.71	3.16	9.14

TSH: thyroid-stimulating hormone; LH: luteinizing hormone; FSH: follicle-stimulating hormone; GH: growth hormone. The basal and highest values of each hormonal axis assessed in the pituitary dynamic tests are shown. ^∗^Altered response to the pituitary dynamic test.
